# Serum Deprivation-Induced Human GM3 Synthase (hST3Gal V) Gene Expression Is Mediated by Runx2 in Human Osteoblastic MG-63 Cells

**DOI:** 10.3390/ijms17010035

**Published:** 2015-12-29

**Authors:** Hyun-Kyoung Yoon, Ji-Won Lee, Kyoung-Sook Kim, Seo-Won Mun, Dong-Hyun Kim, Hyun-Jun Kim, Cheorl-Ho Kim, Young-Choon Lee

**Affiliations:** 1 Department of Medicinal Biotechnology, College of Health Sciences, Dong-A University, Busan 604-714, Korea; gusrud073@naver.com (H.-K.Y.); jwlee0203@dgist.ac.kr (J.-W.L.); kskim@dau.ac.kr (K.-S.M.); ss11033@hanmail.net (S.-W.M.); mose79@dau.ac.kr (D.-H.K.); 2 Department of Orthopaedic Surgery, College of Medicine, Dong-A University, Busan 604-714, Korea; hyeonjun@dau.ac.kr; 3 Molecular and Cellular Glycobiology Unit, Department of Biological Sciences, SungKyunKwan University, Kyunggi-Do 440-746, Korea; chkimbio@skku.edu

**Keywords:** serum deprivation, human GM3 synthase (hST3Gal V), MG-63 cells, runt-related transcription factor 2 (Runx2), transcriptional regulation

## Abstract

Serum deprivation (SD) is well known to induce G0/G1 cell cycle arrest and apoptosis in various cells. In the present study, we firstly found that SD could induce G1 arrest and the differentiation of human osteoblastic MG-63 cells, as evidenced by the increase of osteoblastic differentiation markers, such as bone morphogenetic protein-2 (BMP-2), osteocalcin and runt-related transcription factor 2 (Runx2). In parallel, gene expression of human GM3 synthase (hST3Gal V) catalyzing ganglioside GM3 biosynthesis was upregulated by SD in MG-63 cells. The 5′-flanking region of the hST3Gal V gene was functionally characterized to elucidate transcriptional regulation of hST3Gal V in SD-induced MG-63 cells. Promoter analysis using 5′-deletion constructs of the hST3Gal V gene demonstrated that the −432 to −177 region functions as the SD-inducible promoter. Site-directed mutagenesis revealed that the Runx2 binding sites located side-by-side at positions −232 and −222 are essential for the SD-induced expression of hST3Gal V in MG-63 cells. In addition, the chromatin immunoprecipitation assay also showed that Runx2 specifically binds to the hST3Gal V promoter region containing Runx2 binding sites. These results suggest that SD triggers upregulation of hST3Gal V gene expression through Runx2 activation by BMP signaling in MG-63 cells.

## 1. Introduction

Serum deprivation (SD) has been widely used to produce a synchronized culture by cell cycle arrest in the G0/G1 phase [1,2,3,4,5]. Human glioblastoma T98G and ovarian cancer SK-OV-3 cells were reported to be arrested in G0 and G1, respectively, by SD [6,7]. In addition, it has been demonstrated in numerous studies that the cultured cells, including bone marrow-derived mesenchymal stem cells, undergo apoptosis by SD [8,9,10,11,12,13,14,15]. Moreover, SD induced not only cell differentiation in immortalized rat proximal tubule cells [16], but complete redifferentiation of human umbilical vascular smooth muscle cells [17].

Gangliosides are complex glycosphingolipids bearing sialic acid and, owing to the existence in the external leaflets of plasma membranes of animal cells, they play important roles in cell-cell interaction, adhesion and cell signaling processes [18,19,20,21]. Ganglioside profiles, including its composition and distribution, change dramatically during diverse biological processes, such as cell proliferation, differentiation, development and apoptosis [18,20]. These changes are attributed to the strict regulation of ganglioside biosynthesis by ganglioside synthases, a family of glycosyltransferases, in the Golgi apparatus. It is well known that malignant tumor cells show a different expression pattern of cell surface gangliosides as compared to normal cells, including predominant expression of specific gangliosides [22], which led to the development of antibodies against specific gangliosides as immunotherapeutic agents [23]. Moreover, recent studies have revealed that gangliosides are involved in neuronal and osteoblast differentiation of human mesenchymal stem cells (hMSCs), human adipose-derived stem cells (hADSCs) and human dental pulp-derived stem cells (hDPSCs), as well as mouse embryonic stem cells (mESCs) [24,25,26,27,28]. Gangliosides GM1 and GT1b expression was markedly elevated in the neural differentiation of hMSCs and mESCs [24], whereas GD3 and GD1a were highly expressed in the neural differentiation of hDPSCs [25]. During the differentiation of hMSCs into osteoblasts, reduction of ganglioside GM3 expression was observed, while GD1a expression was remarkably elevated [26,27,28].

On the basis of these observations, we speculated that ganglioside synthesis might be associated with osteoblast differentiation. To confirm this hypothesis, we investigated whether during osteoblast differentiation, gene expression of human ganglioside synthases is regulated in the human osteoblastic MG-63 cells. In this study, we have firstly found osteoblast differentiation by SD and significant elevation of human GM3 synthase (hST3Gal V) gene expression in SD-induced differentiation of MG-63 cells, thereby enhancing ganglioside GM3 expression. To understand the molecular basis of hST3Gal V gene expression induced by SD, furthermore, the promoter region to mediate transcriptional upregulation of hST3Gal V gene in serum-deprived cells was functionally characterized, and we found involvement of bone morphogenetic protein (BMP) BMP/Runx2 signaling in SD-induced hST3Gal V transcriptional activation.

## 2. Results

### 2.1. Effect of Serum Deprivation on Cell Proliferation

**Figure 1:**
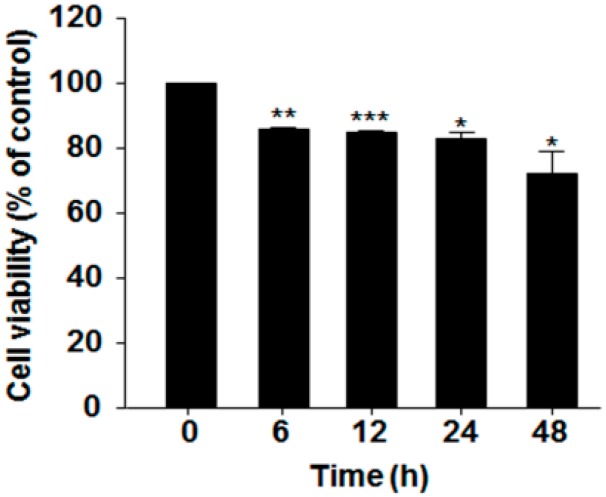
Effect of serum deprivation (SD) on the viability of MG-63 cells. The cytotoxic effects of SD on MG-63 cells were assessed by the MTT assay. Cells were grown in serum-free medium for the indicated time periods, and their absorbance at 540 nm was measured on an enzyme-linked immunosorbent assay (ELISA) reader. The results are expressed as percentages of cell proliferation in the control (0 h) and represent the means ± SEM of three independent experiments. * *p* < 0.05 (compared to the control); ** *p* < 0.01; *** *p* < 0.001.

Prior to investigation of the SD effect on MG-63 cell differentiation and hST3Gal V expression, we first examined the cytotoxicity of SD in MG-63 cells by the tetrazolium salt reduction (MTT) assay. As shown Figure 1, cell viability for a 24-h incubation was more than 80%, and about 73% viability was observed in incubation for 48 h, indicating that MG-63 cell proliferation was not significantly affected under serum-free conditions for 48 h.

### 2.2. Serum Deprivation (SD) Induces G1 Arrest of the Cell Cycle in MG-63 Cells

**Figure 2:**
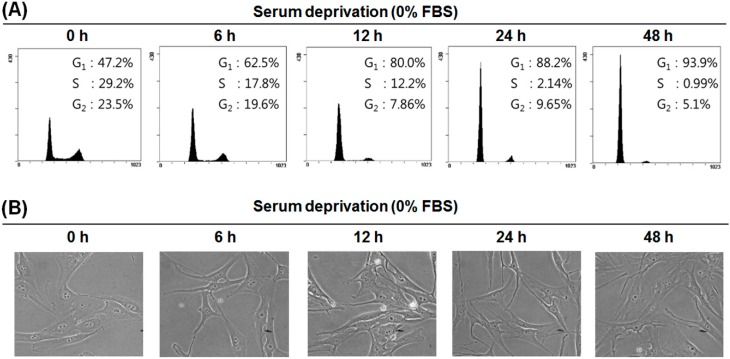
Typical histograms of the DNA content and cell morphology of MG-63 cells cultured under serum-free conditions. Cells were grown in serum-free medium for the indicated time periods. (**A**) DNA content was analyzed by flow cytometry; (**B**) Cell morphological images were taken by phase-contrast microscope (400×) at each time point.

Since the differentiation of mammalian cells is preceded by G1 arrest of the cell cycle [29], we examined whether SD induces G1 arrest of the cell cycle in MG-63 cells. After MG-63 cells were incubated under serum-free conditions for various times, cells were collected, and the cell cycle profile was analyzed by flow cytometry. As shown in Figure 2A, the percentage of cells in the G1 phase was increased time dependently by SD, whereas the percentages of S and G2 phases were decreased. Cells in the sub-G1 phase were not observed, indicating that SD did not cause cell death. In addition, the morphology of MG-63 cells became branched and elongated time dependently (Figure 2B).

### 2.3. Effect of SD on Osteoblast-Related Marker Gene Expression in MG-63 Cells

**Figure 3:**
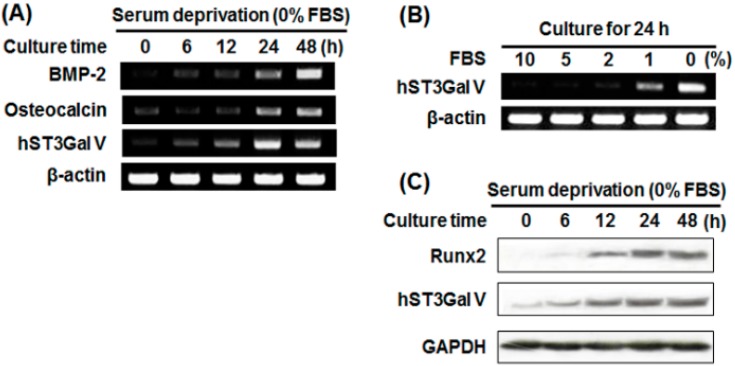
Effect of SD on the expression levels of osteoblastic markers and hST3Gal V. Total RNA from MG-63 cells was isolated after incubation in serum-free medium for the indicated time periods (**A**) or after culture for 24 h in medium containing various concentration of FBS (**B**), and mRNA transcripts of osteoblastic markers and hST3Gal V were detected by reverse transcription-polymerase chain reaction (RT-PCR). As an internal control, parallel reactions were performed to measure the levels of the housekeeping gene β-actin; (**C**) Equal amounts of cell lysates (20 μg) were separated on sodium dodecyl sulfate (SDS)-polyacrylamide gels and transferred to a polyvinylidene fluoride (PVDF) membrane. The membrane was probed with specific antibodies against Runx2 and hST3Gal V. GAPDH was used as an internal control.

**Figure 4:**
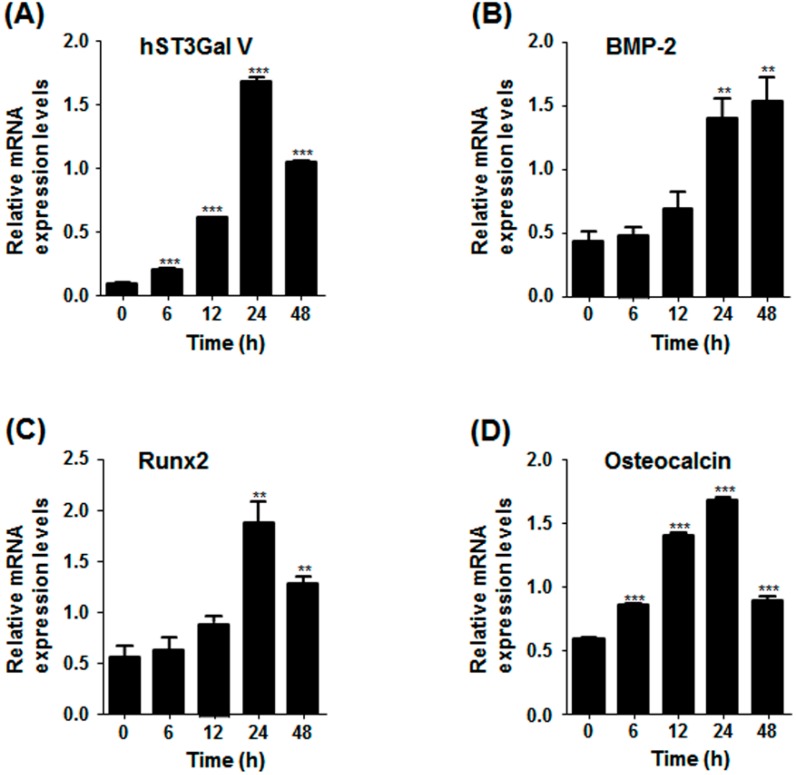
Quantitative real-time PCR analysis of the expression levels of osteoblastic markers and hST3Gal V in SD-induced MG-63 cells. Total RNA from MG-63 cells was isolated after incubation in serum-free medium for the indicated time periods, and mRNA transcripts of hST3Gal V (**A**) and osteoblastic markers (**B**–**D**) were was analyzed by quantitative real-time PCR. The transcript copy numbers of osteoblastic markers and hST3Gal V were normalized to the β-actin transcript copy number for each sample. Experiments were repeated three times to check the reproducibility of results. ** *p* < 0.01 (compared to the control); *** *p* < 0.001.

To investigate whether SD induces the expression of marker genes related to osteoblast differentiation, MG-63 cells were incubated under serum-free conditions for various times. As shown in Figure 3A, SD increased BMP-2 and osteocalcin mRNA levels in a time-dependent manner. In addition, qPCR results showed that mRNA levels of BMP-2, osteocalcin and Runx2 were also enhanced in a time-dependent manner, and peak levels of osteocalcin and Runx2 were reached after SD for 24 h and decreased thereafter (Figure 4). Moreover, the protein levels of Runx2 were increased in proportion to the increment of its mRNA level by SD (Figure 3C). Taken together, these results indicate that SD induces G1 cell cycle arrest and differentiation, but not cell death, in MG-63 cells.

### 2.4. Effect of SD on hST3Gal V Expression in MG-63 Cells

To check whether ganglioside synthesis is associated with osteoblast differentiation, we evaluated the effect of SD on the gene expression of human ganglioside synthases in MG-63 cells. As shown in Figure 3A,B, the mRNA levels of hST3Gal V catalyzing ganglioside GM3 synthesis were markedly increased by SD, and their enhancements were in a time-dependent manner. In addition, these increments were confirmed by qPCR in which the highest level of mRNA expression was observed after SD for 24 h and decreased thereafter (Figure 4). These results clearly revealed that hST3Gal V gene expression was induced by SD.

As the next step, the correlation between mRNA and protein expression levels of hST3Gal V by SD was evaluated by Western blot analysis using an hST3Gal V-specific monoclonal antibody. As shown in Figure 3C, protein levels of hST3Gal V were also increased in a time-dependent manner. These results indicate that the induction of hST3Gal V expression by SD is regulated at both the transcriptional and translational levels.

### 2.5. Effect of SD on Ganglioside GM3 Expression in MG-63 Cells

**Figure 5:**
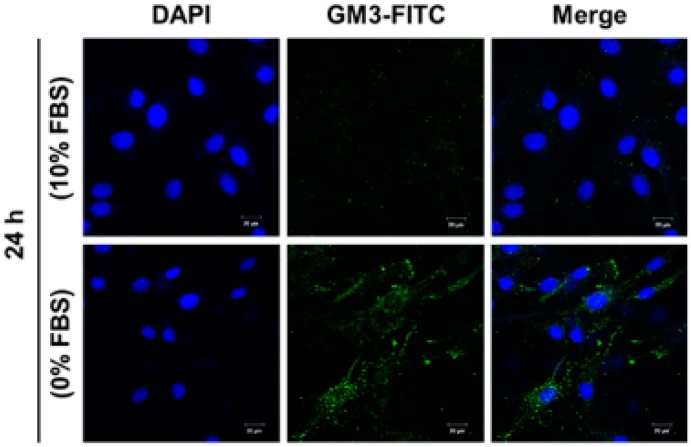
Confocal analysis of ganglioside GM3 expression in SD-induced MG-63 cells. After incubation for 24 h in standard medium containing 10% FBS or 0% FBS, cells were immunostained with anti-GM3 antibodies (FITC; green). Nuclei were stained with DAPI (blue) and analyzed by confocal microscopy. Scale bar: 20 µm.

To check whether or not the increased expression of hST3Gal V by SD leads to the enhancement of ganglioside GM3 level synthesized by hST3Gal V in MG-63 cells, we analyzed the cellular expression level of ganglioside GM3 by immunofluorescence confocal microscopy using the mouse anti-GM3 monoclonal antibody and fluorescein isothiocyanate (FITC)-conjugated goat-anti-mouse IgM as the secondary antibody to visualize GM3 expression induced by SD in MG-63 cells. As shown in Figure 5, the expression level of ganglioside GM3 was increased in MG-63 cells incubated in serum-free medium for 24 h, but not in MG-63 cells incubated in 10% FBS-containing medium.

### 2.6. Analysis of Transcriptional Activity of the hST3Gal V Promoter by SD in MG-63 Cells

**Figure 6:**
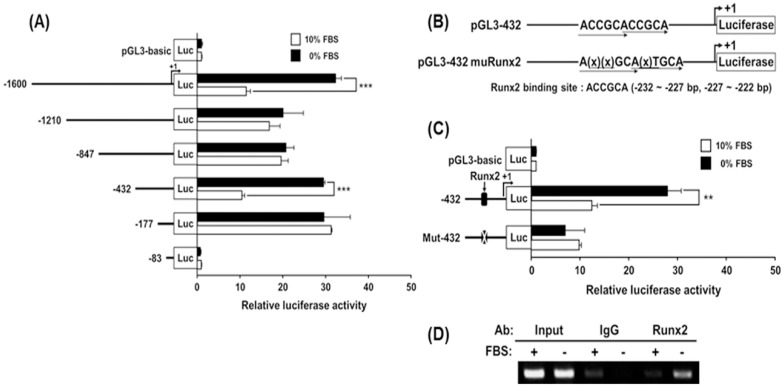
Analysis of the hST3Gal V promoter activity in SD-induced MG-63 cells. The schematic diagrams represent DNA constructs (**A**) containing various lengths of the wild-type hST3Gal V promoter or constructs (**B**,**C**) with the mutant Runx2 sequence in the 5′-flanking region, upstream of a luciferase reporter gene; the transcription start site is designated +1. The pGL3-basic construct, which did not contain a promoter or an enhancer, was used as a negative control. Each construct was transfected into MG-63 cells, with pRL-TK co-transfected as an internal control. The transfected cells were incubated in the presence (open bar) or absence (solid bar) of 10% FBS for 24 h. Relative firefly luciferase activity was measured using the Dual-Luciferase Reporter Assay System, and all firefly activity was normalized to the *Renilla* luciferase activity derived from pRL-TK. The values represent the means ± SD of three independent experiments with triplicate measurements. ** *p* < 0.01; *** *p* < 0.001; (**D**) PCR amplification in the −432 and −177 region of the hST3Gal V promoter on chromatin immunoprecipitated with either an antibody against Runx2 or control IgG from MG-63 cells grown in the presence or absence of 10% FBS for 24 h. The input (10-fold diluted) represents the positive control.

Since the mRNA transcript levels of hST3Gal V were significantly increased by SD in MG-63 cells (Figure 3 and Figure 4), using the luciferase reporter gene assay system, the transcriptional activity of the hST3Gal V promoter was evaluated to clarify whether the transcriptional activity of hST3Gal V is controlled in SD-induced MG-63 cells. As shown in Figure 6A, a markedly enhanced luciferase activity was observed in cells harboring the pGL3-432 construct under serum-free conditions for 24 h, about 3.0-fold higher than cells incubated in 10% FBS-containing medium. However, SD in cells carrying other promoter constructs and the pGL3-basic (negative control) did not trigger the remarkable increase of their luciferase activities. These data suggest that the region between −432 and −178 is indispensable for the SD-responsive promoter function of hST3Gal V in MG-63 cells.

### 2.7. Identification of the SD-Responsive Element in the Functional −432/−178 Region of the hST3Gal V Promoter

To identify the SD-responsive element in the nucleotide −432 to −178 region of the hST3Gal V gene, based on the above results, we firstly focused on transcription factor Runx2, which is essential for osteoblast differentiation and is expressed in all osteoblasts [30]. By DNA sequence analysis, we found two potential Runx2 binding sites in the promoter region between −432 and −177 of the hST3Gal V gene. Two are located side-by-side at positions −232 and −222 in the hST3Gal V gene (Figure 6C). These DNA sequences, ACCGCACCGCA, closely match the consensus Runx binding motif, 5′-PuACCPuCA-3′ [31]. To clarify whether these binding sites are closely associated with SD-induced expression of hST3Gal V in MG-63 cells, one mutant plasmid (pGL3-432muRunx2) was constructed (Figure 6B) and then transfected into MG-63 cells, and luciferase assays were performed. As shown in Figure 6C, pGL3-432muRunx2 remarkably decreased transcriptional activity to more than two-fold of pGL3-432. This result indicates that these Runx2-binding sites are indispensable for the SD-induced expression of hST3Gal V. To further confirm whether Runx2 could bind to these sequences in the hST3Gal V promoter, we used the chromatin immunoprecipitation (ChIP) assay to exam the *in vivo* association of Runx2 with promoter region between −432 and −177 of the hST3Gal V gene in SD-induced MG-63 cells. As shown Figure 6D, as the specific PCR products obtained with input DNA, Runx2-specific amplification by PCR was observed in MG-63 cells incubated under serum-free medium for 24 h to induce the expression of the hST3Gal V gene. However, distinct binding in a control assay with 10% FBS or IgG antibody was not observed. These results indicate that the hST3Gal V gene expression in SD-induced MG-63 cells is upregulated by direct binding of Runx2 to the hST3Gal V promoter region containing Runx2 binding sites.

## 3. Discussion

After the promoter region of hST3Gal V was firstly isolated and characterized by our group [32], we became interested in investigating the mechanisms controlling hST3Gal V gene expression [33,34,35,36,37,38]. In previous studies, we have shown that the consensus CREB binding site (TGACGTCA) at position −143 to −136 in the hST3Gal V gene contributes to transcriptional activation of hST3Gal V during monocytic differentiation of HL-60 cells [33] and megakaryocytic differentiation of K562 cells induced by phorbol 12-myristate 13-acetate (PMA) [34]. In addition, we have demonstrated that the hST3Gal V gene expression is upregulated through a PKC/ERK/CREB-dependent pathway during the differentiation of PMA-induced HL-60 cells [35]. Moreover, we have also revealed that this CREB binding site is crucial for the transcriptional activity of hST3Gal V in valproic acid-induced SK-N-BE(2)-C human neuroblastoma cells [36] and ARPE-19 human retinal pigment epithelial cells [37]. In the present study, we have demonstrated for the first time that hST3Gal V expression was upregulated during osteoblast differentiation induced by SD, and two potential Runx2-binding sites at positions −232 and −222 in the hST3Gal V gene play a critical role in transcriptional activation of hST3Gal V at the time.

Although cell differentiation and redifferentiation induced by SD has been reported in immortalized rat proximal tubule cells [16] and human umbilical vascular smooth muscle cells, respectively [17], SD-induced osteoblast differentiation is not known to date. Here, we firstly report osteoblast differentiation by SD using the human osteoblastic cell line MG63. The present study provided evidence that SD induces G1 arrest of the cell cycle in MG-63 cells, as revealed by flow cytometry analysis. Moreover, this study demonstrated for the first time that SD enhanced significantly the transcript levels of marker genes of osteoblast differentiation, such as BMP, osteocalcin and Runx2, as evidenced by RT-PCR and qPCR. Especially, the temporal relationship between G1 arrest and the increase in the levels of both Runx2 mRNA and protein in SD-induced MG63-cells is coincident with the previous report [39] that showed the cell cycle G1 arrest concomitant with upregulation of both Runx2 mRNA and protein in response to SD in immortalized mouse MC3T3 osteoblastic cells. Given that G1 arrest of the cell cycle precedes the differentiation of most mammalian cells [29] and cellular differentiation requires the coordination of G1 cell cycle arrest and cell-specific gene expression [40], our results suggest that SD induced osteoblastic differentiation in MG-63 cells.

In the current study, we also demonstrated that SD triggers a significant time-dependent enhancement in both mRNA and protein levels of hST3Gal V, as shown in RT-PCR, qPCR and Western blot analysis, suggesting that SD induces upregulation of hST3Gal V in transcriptional and translational levels. In addition, the increased hST3Gal V expression was accompanied by the remarkable increment of ganglioside GM3 in SD-induced MG-63 cells, as evidenced by immunostaining with GM3 monoclonal antibody. These data suggest the close temporal relation between hST3Gal V gene expression and GM3 production in SD-induced MG-63 cells. On the other hand, the mRNA and protein levels of hST3Gal V showed increases at earlier time points than BMP-2 and Runx2, respectively, suggesting that there may be other regulatory mechanisms than the BMP-Runx2-dependent transcriptional regulation of hST3Gal V. We also identified the SD-responsive core promoter region between −432 and −178 of the hST3Gal V gene by deletion mutant analysis. This region is the same as that of the core promoter essential for the transcriptional activation of the hST3Gal V in transforming growth factor (TGF)-β1-induced human lens epithelial (HLE) B-3 cells [38]. In our recent study, we clarified that two Sp1-binding sites in this region play an important role in the transcriptional activation of the hST3Gal V in TGF-β1-induced HLE B-3 cells [38]. In the present study, on the other hand, we firstly verified that two Runx2-binding sites in this region are crucial for the transcriptional activation of the hST3Gal V in SD-induced MG-63 cells, as demonstrated by site-directed mutagenesis experiments and the *in vivo* ChIP assay. A previous study has shown that there are three Runx2-binding sites (−602, −1151 and −1834) in the promoter region of the mouse ST3Gal V gene and that by the ChIP assay, Runx2 binds to these sites in murine NIH3T3 fibroblast-derived cells expressing the *Runx2* gene [41]. However, which Runx2-binding site is the major site for transcriptional activation of the mouse ST3Gal V gene remains unclear.

Runx2, a downstream target of BMP, is a transcription factor that induces osteoblast-specific gene expression and thereby plays an essential role in osteoblast differentiation [42,43]. Runx2-binding sites exist in the promoter of marker genes associated with osteoblastic cell differentiation, such as *osteocalcin gene 1* (*OG1*), *a1(I) collagen*, *bone sialoprotein* (*Bsp*) and *osteopontin*, and therefore, osteoblast-specific expression of these genes is known to be dependent on Runx2 [30]. It is also known that multiple Runx2-binding sites are contained in the promoter region of the Runx2 gene, and thus, Runx2 regulates positively or negatively its own promoter activity [44]. It is well established that Runx2 is transcriptionally upregulated by BMP, which triggers the cascade of events responsible for osteoblast differentiation, and the interaction of Runx2 with Smad 1 and 5 is crucial for the enhancement of the transcriptional activity of Runx2 by BMP signaling [31,40,41,42,43,44].

Although the DNA binding and transcriptional activity of Runx2 may be controlled by the MAPK pathway or PI3K-Akt signaling [42], in this study, the functionality of Runx2 responsible for the transcriptional activation of the hST3Gal V in SD-induced MG-63 cells might be regulated by BMP signaling, because BMP and Runx2 were simultaneously expressed in MG-63 cells incubated under serum-free conditions for 24 h. Given that the BMP signal cascade starts out with the activation of Smad 1/5/8 by phosphorylation, followed by the complex formation with Smad 4 and then translocation into the nucleus where, they trigger the expression of target genes, including Runx2 [43,44], further study is required to clarify the functionality of Runx2 by interaction with the activated Smad proteins leading to a transcriptional upregulation of hST3Gal in SD-induced MG-63 cells.

## 4. Experimental Section

### 4.1. Cell Cultures

Human osteoblastic cell line MG-63 obtained from American Type Culture Collection (Manassas, VA, USA) was cultured in Dulbecco’s Modified Eagle’s Medium (DMEM; WelGENE Co., Daegu, Korea) at 37 °C in a 5% CO_2_ incubator. Medium was supplemented with 100 U/mL of penicillin, 100 μg/mL of streptomycin and 10% (*v*/*v*) fetal bovine serum (FBS) (Gibco BRL, Life Technologies; Grand Island, NY, USA). To induce cell differentiation and the increased expression of the hST3Gal V gene, the cell was placed in serum-free medium for various time periods.

### 4.2. Cell Viability Assay

To analyze cell viability, cells were plated in 24-well culture plate (5.0 × 10^4^ cells/well). After 24 h, the medium was replaced with serum-free medium and then incubated for various times. Cell viability was determined by the MTT assay, as described previously [45]. The amount of formazan salt was measured by absorbance at 540 nm using an enzyme-linked immunosorbent assay (ELISA) plate reader (Bio-Rad, Hercules, CA, USA). Cell viability was quantified as a percentage relative to the control.

### 4.3. Reverse Transcription-Polymerase Chain Reaction and Quantitative Real-Time PCR

Total RNA was extracted from cultured cells using TRIzol-Reagent (Invitrogen; Carlsbad, CA, USA) following the manufacturer’s instructions, and first-strand cDNA synthesis was done using RNA to cDNA EcoDry TM Premix (Oligo dT) kit (Clontech 639543). The synthesized cDNA was amplified by PCR with specific hST3Gal V, bone morphogenic protein-2 (BMP-2), osteocalcin and β-actin primers, shown in Table 1. PCR amplification was carried out using a PC-818A Program Temp Control System (Astec, Fukuoka, Japan), with 1 cycle at 95 °C for 5 min and 28 cycles consisting of denaturation for 40 s at 95 °C, annealing for 40 s at specific annealing temperatures for the primers (Table 1) and extension at 72 °C for 45 s, followed by 7 min at 72 °C. PCR products were analyzed by 1% agarose gel electrophoresis. Real-time qRT-PCR was performed using a CFX96^TM^ Real-Time system with SYBR Premix (Bio-Rad) and the specific primers as shown in Table 1. Relative quantitation was conducted using CFX Manager v2.1 software (Bio-Rad). The amplification program of quantitative real-time PCR was composed of 1 cycle at 95 °C for 3 min, followed by 45 cycles with a denaturing step at 95 °C for 10 s, an annealing step of 20 s at specific annealing temperatures for the primers (Table 1) and an elongation step at 72 °C for 20 s. Target gene expression was normalized to β-actin mRNA expression.

**Table 1:** Primer sequences used for reverse transcription-polymerase chain reaction (RT-PCR) and quantitative real-time PCR (qPCR), site-directed mutagenesis and chromatin immunoprecipitation (ChIP).

Primer	Sequence	Strand	Purpose
hST3Gal V	5′-CCCTGCCATTCTGGGTACGAC-3′	Sense	RT-PCR
hST3Gal V	5′-CACGATCAATGCCTCCACTGAGA-3′	Antisense	RT-PCR
BMP-2	5′-ATGTTCGCCTGAAACAGAGACCCA-3′	Sense	RT-PCR
BMP-2	5′-CTTACAGCTGGACTTAAGGCGTTTC-3′	Antisense	RT-PCR
Osteocalcin	5’-ATGAGAGCCCTCACACTCCTC-3′	Sense	RT-PCR
Osteocalcin	5′-GCCGTAGAAGCGCCGATAGGC-3′	Antisense	RT-PCR
β-actin	5′-CAAGAGATGGCCACGGCTGCT-3′	Sense	RT-PCR
β-actin	5′-TCCTTCTGCATCCTGTCGGCA-3′	Antisense	RT-PCR
hST3Gal V	5′-GAACTCTTGCCAGAGCACGA-3′	Sense	qRT-PCR
hST3Gal V	5′-CCCAGTTCTAATCCGTGCAG-3′	Antisense	qRT-PCR
BMP-2	5′-GGGTTGGAACTCCAGACTGT-3′	Sense	qRT-PCR
BMP-2	5′-GAAGAGTGAGTGGACCCCAG-3′	Antisense	qRT-PCR
Osteocalcin	5′-GAGGGCAGCGAGGTAGTGAA-3′	Sense	qRT-PCR
Osteocalcin	5′-GGCTCCCAGCCATTGATACA-3′	Antisense	qRT-PCR
ALP	5′-CCACGTCTTCACATTTGGTG-3′	Sense	qRT-PCR
ALP	5′-AGACTGCGCCTAGTAGTTGT-3′	Antisense	qRT-PCR
Runx2	5′-CCAGATGGGACTGTGGTTACTG-3′	Sense	qRT-PCR
Runx2	5′-CGGAGCTCAGCAGAATAATTTTC-3′	Antisense	qRT-PCR
β-actin	5′-ACCCACTCCTCCACCTTTGAC-3′	Sense	qRT-PCR
β-actin	5′-CCTGTTGCTGTAGCCAAATTCG-3′	Antisense	qRT-PCR
muRunx2	5′-CTCTGGCAATGCCC*AGCATGCA*GGGCTGACTGGCCG-3′	Sense	Mutagenesis
muRunx2	5′-CGGCCAGTCAGCCC*TGCATGCT*GGGCATTGCCAGAG-3′	Antisense	Mutagenesis
hST3Gal V	5′-GCCCCGGGTGCGTCCCTG-3′	Sense	ChIP
hST3Gal V	5′-AGCGCCGCTCTCGCGCC-3′	Antisense	ChIP

The mutated nucleotides in the oligonucleotides for mutation are underlined and in italics.

### 4.4. Site-Directed Mutagenesis

pGL3-432 mutRunx2 with base deletion at binding sites of the runt-related transcription factor 2 (Runx2) were constructed using a QuikChange® II XL site-directed mutagenesis kit (Stratagene, La Jolla, CA, USA) according to the manufacturer’s protocol using the oligonucleotide primers (Table 1). The presence of mutations in the Runx2-binding sites was confirmed by DNA sequencing.

### 4.5. Transfection and Luciferase Assay

The luciferase reporter plasmids utilized herein, pGL3-1600, and its derivatives (pGL-83 to pGL3-1210) have been reported elsewhere [32,33,34,35,36,37,38]. To study the effect of SD on hST3Gal l V promoter activity, cells were seeded at a density of 5.0 × 10^4^ cells/well into 24-well culture plate and, after incubating for 15 h, co-transfected with 0.5 μg of luciferase reporter plasmid and 50 ng of pRL-TK as the control *Renilla* luciferase vector (Promega; Madison, WI, USA), using 1 μL Lipofectamine 2000 (Invitrogen). After 18 h of recovery in normal medium with serum, the medium was replaced with serum-free medium. After culture for an additional 24 h in serum-free medium, cells were harvested and assayed using the Dual-Luciferase Reporter Assay System (Promega) and a GloMax™ 20/20 luminometer (Promega).

### 4.6. Immunofluorescence

Immunofluorescence staining was performed as previously described [45]. MG-63 cells grown in serum-free medium for 24 h were fixed with 3% paraformaldehyde for 10 min at 37 °C, washed three times with PBS and blocked with 1% BSA for 1 h at 37 °C. After incubation for overnight at 4 °C with the anti-GM3 monoclonal antibody (mouse IgM, Kappa-chain, clone: GMR6; Seigakagu; Japan), cells were reacted with FITC-conjugated goat-anti-mouse IgM (Sigma; St. Louis, MO, USA) used as the secondary antibody for 1 h at 37 °C. The nucleus was stained with DAPI for 5 min at 37 °C. Cells were imaged with an LSM 700 confocal laser scanning microscope (Carl Zeiss, Oberkochen, Germany).

### 4.7. Western Blot Analysis

Western blot analysis was performed as previously described [45]. Cells were lysed in RIPA buffer, and the cell lysate was resolved in SDS-PAGE and then transferred to a PVDF membrane. The membranes were incubated sequentially with primary and secondary antibodies. Blots were detected using the ECL chemiluminescence system (GE Healthcare, Piscataway, NJ, USA). The following primary antibodies were used: Runx2 (M-70, Santa Cruz, CA, USA); GM3 synthase (H-125, Santa Cruz, CA, USA) and GAPDH (#MAB374, Millipore, Milford, MA, USA). Horseradish peroxidase (HRP)-conjugated secondary antibodies were obtained from Enzo Life Science (Farmingdale, NY, USA).

### 4.8. Chromatin Immunoprecipitation Assay

The ChIP assay was carried out using the ChIP kit (Upstate Biotechnology, Syracuse, NY, USA) according to the manufacture’s instruction. As described previously [37], cells were cross linked in 1% formaldehyde at 37 °C for 10 min to cross-link DNAs and protein and then sonicated to shear the DNAs. Immunoprecipitation was performed using 10 µg of Runx2 (M-70, Santa Cruz, CA, USA) and IgG antibodies (Sigma; St. Louis, MO, USA). The purified ChIP DNA or input DNA was used for PCR analysis using the primer flanking Runx2 binding site on the hST3Gal V promoter (Table 1).

### 4.9. Flow Cytometry Analysis

To analyze DNA content, cells were plated in 24-well culture plates (5.0 × 10^4^ cells/well). After 24 h, the medium was replaced with serum-free medium and then incubated for various times. The cells were collected, fixed with 70% ethanol and suspended in PBS containing 0.5 mg/mL propidium iodide, 0.5 mg/mL RNase and 0.03% NP-40. After incubation in the dark for 30 min at room temperature, the cells were analyzed by a Beckman-Coulter Cytomics FC500 flow cytometer equipped with CXP software (Beckman-Coulter, Miami, FL, USA).

## 5. Conclusions

In this study, we showed for the first time that the expression of hST3Gal V mRNA is upregulated during the differentiation of human osteoblastic MG-63 cells induced by SD. Furthermore, we demonstrated that hST3Gal V and ganglioside GM3 expressions are also markedly increased in SD-induced MG-63 cells. Although the precise mechanisms involved in the SD-triggered activation of Runx2 leading to the transcriptional upregulation of hST3Gal V are unknown, we have demonstrated here for the first time that SD induces the enhanced expression of hST3Gal V gene and subsequent accumulation of ganglioside GM3 levels through through Runx2 activation by BMP signalling in MG-63 cells.
